# Ecological approaches in veterinary epidemiology: mapping the risk of bat-borne rabies using vegetation indices and night-time light satellite imagery

**DOI:** 10.1186/s13567-015-0235-7

**Published:** 2015-09-04

**Authors:** Luis E Escobar, A Townsend Peterson, Monica Papeş, Myriam Favi, Veronica Yung, Olivier Restif, Huijie Qiao, Gonzalo Medina-Vogel

**Affiliations:** Facultad de Ecología y Recursos Naturales, Universidad Andres Bello, Av. República 440, Santiago, Chile; Center for Global Health and Translational Science, SUNY Upstate Medical University, Syracuse, New York USA; Biodiversity Institute, University of Kansas, Lawrence, Kansas USA; Department of Integrative Biology, Oklahoma State University, Stillwater, Oklahoma 74078 USA; Sección Rabia, Instituto de Salud Publica de Chile, Av. Maraton 1000, Ñuñoa, Chile; Disease Dynamics Unit, Department of Veterinary Medicine, University of Cambridge, Madingley Road, Cambridge, CB3 0ES UK; Key Laboratory of Animal Ecology and Conservation Biology, Institute of Zoology, Chinese Academy of Science, Beijing, China

## Abstract

Rabies remains a disease of significant public health concern. In the Americas, bats are an important source of rabies for pets, livestock, and humans. For effective rabies control and prevention, identifying potential areas for disease occurrence is critical to guide future research, inform public health policies, and design interventions. To anticipate zoonotic infectious diseases distribution at coarse scale, veterinary epidemiology needs to advance via exploring current geographic ecology tools and data using a biological approach. We analyzed bat-borne rabies reports in Chile from 2002 to 2012 to establish associations between rabies occurrence and environmental factors to generate an ecological niche model (ENM). The main rabies reservoir in Chile is the bat species *Tadarida brasiliensis*; we mapped 726 occurrences of rabies virus variant AgV4 in this bat species and integrated them with contemporary Normalized Difference Vegetation Index (NDVI) data from the Moderate Resolution Imaging Spectroradiometer (MODIS). The correct prediction of areas with rabies in bats and the reliable anticipation of human rabies in our study illustrate the usefulness of ENM for mapping rabies and other zoonotic pathogens. Additionally, we highlight critical issues with selection of environmental variables, methods for model validation, and consideration of sampling bias. Indeed, models with weak or incorrect validation approaches should be interpreted with caution. In conclusion, ecological niche modeling applications for mapping disease risk at coarse geographic scales have a promising future, especially with refinement and enrichment of models with additional information, such as night-time light data, which increased substantially the model’s ability to anticipate human rabies.

**Electronic supplementary material:**

The online version of this article (doi:10.1186/s13567-015-0235-7) contains supplementary material, which is available to authorized users.

## Introduction

In light of its continued threat to public health around the world, rabies has stimulated considerable research efforts for the development of techniques and tools for vaccination and diagnosis [1]. While rabies control in dogs remains the priority in Africa and Asia [2], the management of wildlife reservoirs is the major challenge for rabies control in the Americas [3,4]. Thanks to rabies-elimination efforts in some American countries, there has been recent progress in acquiring data on rabies in wildlife over large areas [5], but more research is needed to improve spatiotemporal predictions of rabies spillover and guide government interventions.

Chile has a long history of rabies management and control [6]. Improvements in vaccination campaigns, diagnosis, surveillance, data management, and education have served to eradicate dog-related rabies in the country, with consequent reduction of human cases [7,8]. At present, Chile has only a sylvatic cycle of rabies in bats [9]. The main reservoir identified in Chile is the insectivorous bat *Tadarida brasiliensis* [8], a synanthropic bat species with a geographic distribution ranging from Canada to southern South America [10]. With increasing reports of cases of bat-borne rabies in Chile in recent years, a detailed risk map is needed urgently [8]. The Instituto de Salud Pública (ISP) Rabies Laboratory in Chile keeps records of all cases since 1929 [6], including diagnosis methods and detailed geographic location, collected mostly through a national passive surveillance system, which could be the basis for detailed mapping.

Spatial epidemiology is an emerging subdiscipline of epidemiology that aims to identify geographic areas with elevated risk of disease transmission [11], whereas environmental epidemiology works in parallel to identify environmental factors linked to disease appearance. Ecological niche modeling (ENM) can be a useful tool in reaching the goals of both fields: the resulting models allow researchers to estimate environmental factors that shape spatial distributions of organisms [12]. This integration of ecology and biogeography into public health and epidemiology allows understanding the geography of past, current, and emerging disease transmission [13,14], and explaining the role of environmental changes on climate and landscape [15].

Here, we evaluate ENM performance in predicting rabies cases across Chile using environmental variables from satellite imagery to generate high-resolution maps of rabies’ potential distribution across Chile. First, we evaluated the ability of ENM to predict rabies cases across space and time, using a series of environmental variables, geographic regions, and time periods to calibrate and evaluate model predictions. Second, we mapped rabies’ potential distribution across Chile based on environmental variation in a vegetation greenness index derived from Moderate Resolution Imaging Spectroradiometer (MODIS) satellite imagery. Our results demonstrate the usefulness of ENM for the management of zoonotic diseases. In particular, this method could be employed to quantify effects of land-use change on disease emergence and anticipate disease transmission in areas with lack of surveillance.

## Materials and methods

**Figure 1:**
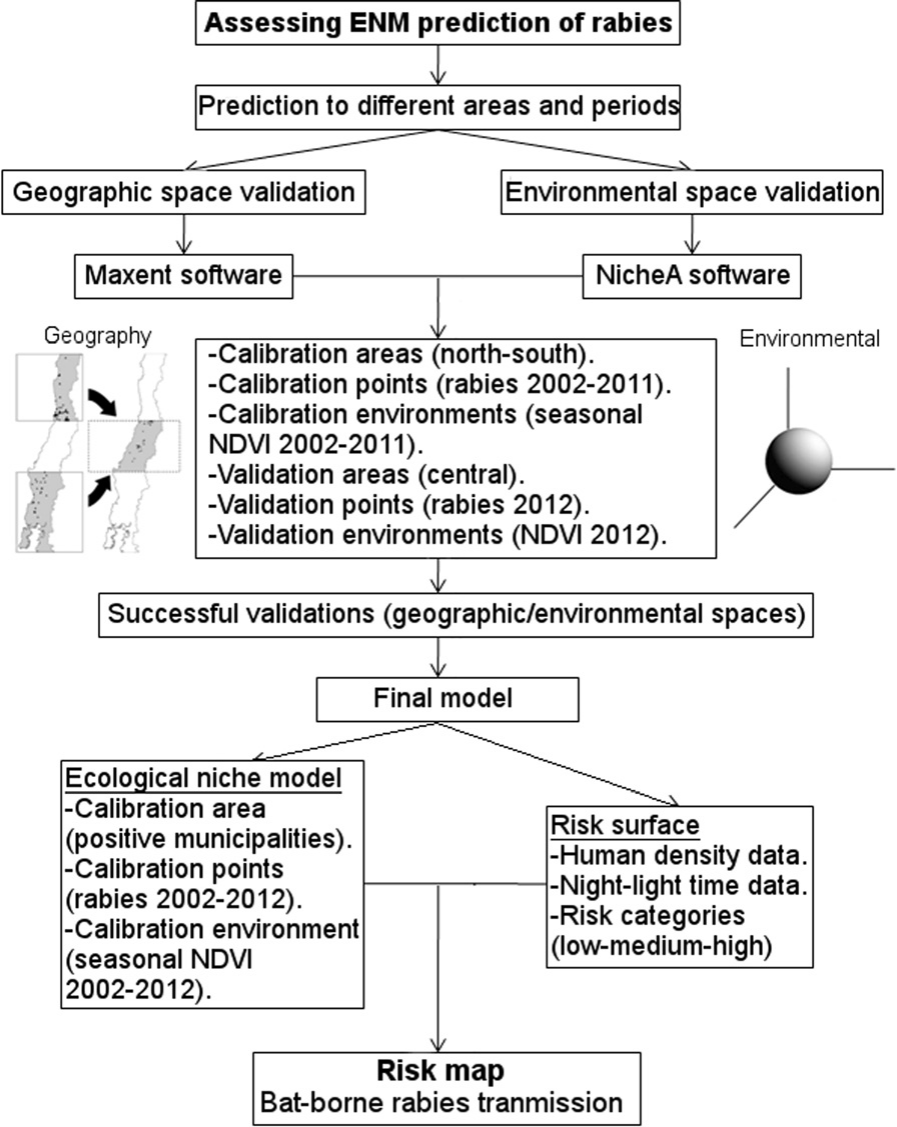
**Flow chart of the use of ecological niche modeling for mapping bat-borne rabies risk.** Before generating the final model, the robustness of the use of NDVI, ecological niche modeling algorithm, and prediction to different areas and periods were assessed in the geographic and environmental space.

First, we assessed the usefulness of ENM to forecast bat-borne rabies using validation metrics in the geographic and the environmental spaces; once the informative capacity of environmental variables and occurrences was corroborated, we developed a final model with a post-processing step to include risk categories (Figure 1).

### Study area

**Figure 2:**
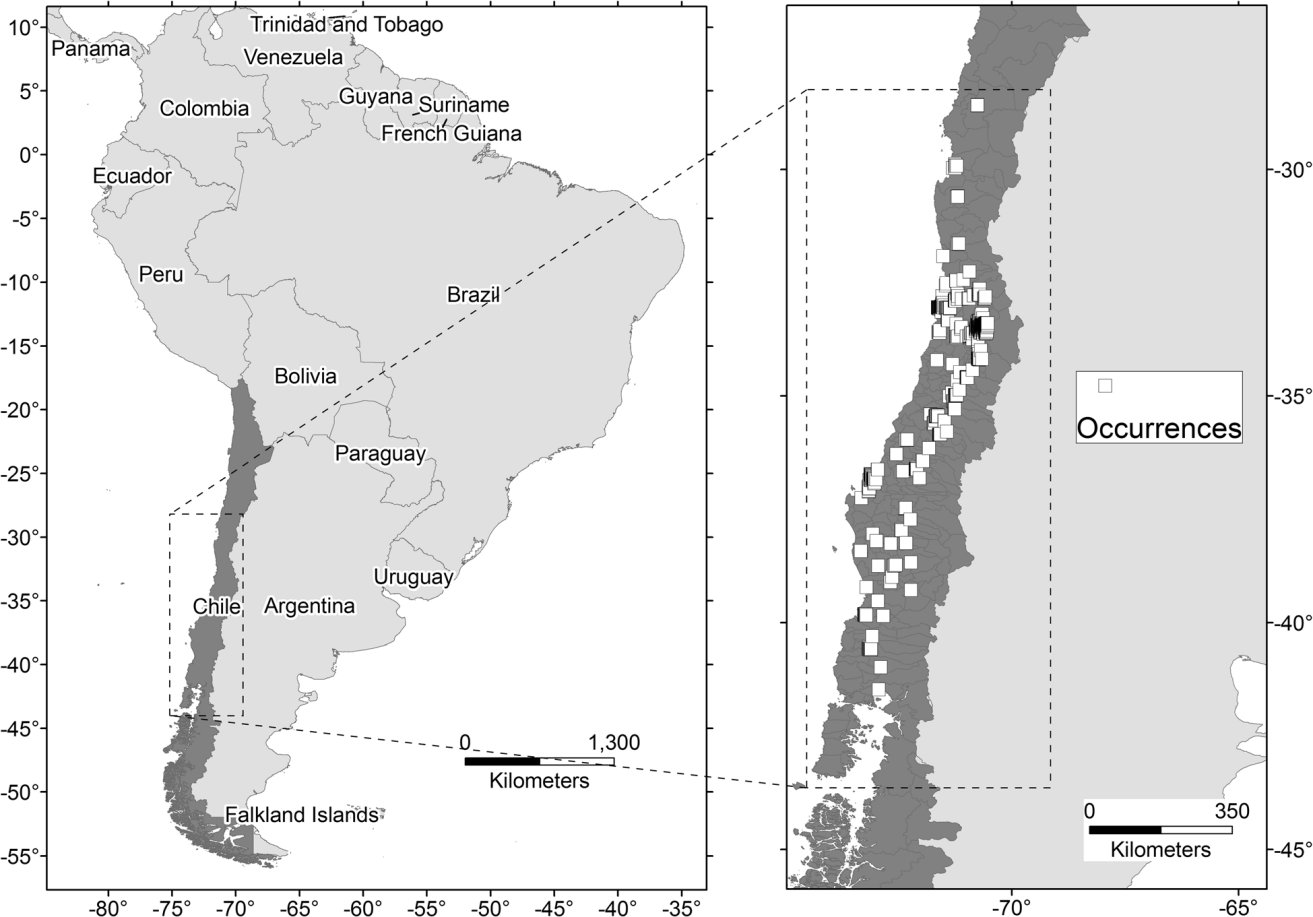
**Study areas for model calibration and validation (M).** Left: geographic position of Chile (dark gray). Right: study areas within central Chile (dark gray) and occurrence points (white squares) in the calibration area (dashed line).

Considering the critical role of the extent of the area of analysis in ENM performance [16], we limited the area for model calibration based on biogeographic barriers that included the Andes mountains (East), Pacific Ocean (West), ice fields in Patagonia (South), and the Atacama Desert (North; Figure 2). This area contained central Chile (43.5° S - 28.0° S) and was our *a priori* hypothesis regarding the extent of the accessible area, or **M** [16,17].

### Input data

Bat-rabies occurrences during 2002–2012 were obtained from samples submitted for rabies testing to ISP from passive and active surveillance. Passive surveillance is the term used for bats submitted by individuals after accidental findings of sick or dead animals, while active surveillance represents bat sampling developed by staff from the Ministry of Health in response to complaints of bats presence. Rabies diagnosis was based on direct immunofluorescence tests on bat brain tissues to confirm virus presence [18]. Identification of monoclonal antibodies and virus genotypes was carried out on positive samples [19-21]. Positive records were georeferenced using the freely available Address Validation Tool to convert textual addresses and location details into latitude and longitude coordinates with ~ 8 m error; sites with less accurate details (e.g., municipal parks) were identified using GoogleEarth with an estimated error <500 m. In all, of 870 positive bat records, 813 samples from passive (98%) and active surveillance (2%) had address information sufficiently detailed for georeferencing. When multiple occurrences fell in the same grid cell, duplicates were removed, leaving single occurrences per cell. The final dataset included 726 occurrences (Figure 2).

As a source of environmental information, we used vegetation index data from MODIS satellite imagery. The most important features of vegetation index datasets are their relationship to primary productivity and the fine (8–16 day composites) temporal resolution [22]. We used the Normalized Difference Vegetation Index (NDVI), as this index provides a measure of herbaceous biomass and physiology through a ratio of light reflected in the red (R) and near-infrared (NIR) spectral bands [23]. This index is calculated as (NIR-R)/(NIR + R), approximating levels of photosynthetic activity, and has been associated with animal distribution and abundance in previous studies [24].

We used 16-day composites of NDVI data: data sets summarizing bi-weekly NDVI values, at ~500 m resolution, considering our accuracy in occurrences location, were downloaded for 2002–2012 from US Geological Survey ([25]; MOD13A1.005). The original files in Hierarchical Data Format-EOS (HDF), with sinusoidal projection, were converted to GeoTIFF (Tagged Image File Format) with geographic projection using the MODIS Reprojection Tool provided by the NASA Goddard Space Flight Center [26]. NDVI values range from −2000 to 10 000, with fill values (No Data) set to −3000. NDVI layers were converted to ASCII files, and fill values changed from −3000 to −9999 to match modeling software requirements. No control quality flag layers were employed. Additionally, we included elevation information from the NASA Shuttle Radar Topographic Mission (SRTM) at ~500 m resolution [27]. The importance of parameters in explaining known cases of rabies was assessed before generating the final model (see Figure 1).

### Model calibration and validation (geographic space)

**Figure 3:**
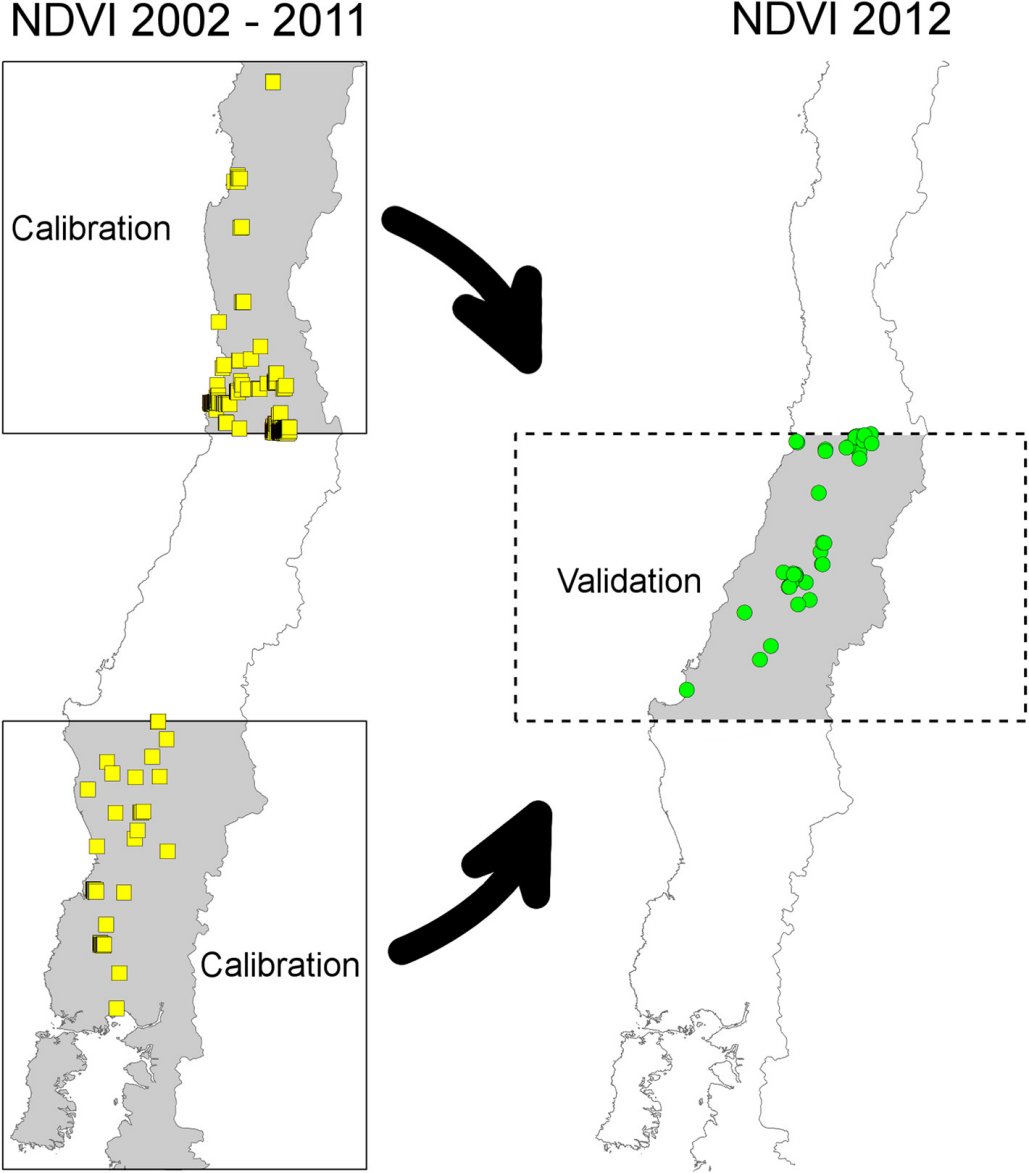
**Model validation schema followed in this study.** Calibration areas (black line boxes, left panel) contain occurrences from 2002 to 2011 (yellow squares, left panel) used to predict rabies in 2012 (green points, right panel) in the validation area (dashed box, right panel). Left panel: calibration areas based on NDVI layers and occurrences from 2002 to 2011. Right panel: validation area based NDVI layers and occurrences for 2012.

We designed a first experiment by calibrating the model with rabies occurrences and NDVI data from 2002–2011, and validating with data from 2012, representing distinct regions in geographic space (Figure 3). Areas for calibration and validation corresponded to three regions of equal latitudinal width (Figure 3), of which we used northern and southern sectors for calibration, and the central region for validation. This framework allowed us to evaluate model performance across space and time. For this modeling experiment, we averaged 16-day NDVI values by season. Seasons were categorized as Summer for NDVI values between 15 December – 15 February, Autumn 15 March – 15 May, Winter 15 June – 15 August, and Spring 15 September – 15 November. Inter-seasonal NDVI datasets were discarded, as they represented transitional values between seasons. Minimum, mean, and maximum seasonal values were calculated for validation and calibration areas, and all 12 environmental layers (four seasons x three summary statistics) were used in model calibration. The jackknife model accuracy gain test in Maxent using all occurrences identified the contribution of each environmental variable to model performance across the calibration area.

Models were generated using Maxent software version 3.3.3.k [28]. Maxent determines the probability distribution of maximum entropy (the most uniform), but constrained to the expected environmental values that correspond to the occurrence records [28]. Maxent is similar to a logistic regression algorithm for presence-background data (i.e., no true absences available), associating occurrences (presence) with environmental data across the study area (background). We selected the following settings in Maxent: random seed with 20% of occurrences set aside to evaluate models, 10 bootstrap permutations, logistic output, and the median of permutations as output. Additionally, models were calibrated with clamping and extrapolation options turned off [29]. Using ESRI ArcGIS 9.3, continuous output models from Maxent were converted to binary maps using a threshold based on omission error, finding the highest Maxent value that omitted no more than 5% of points employed during model calibration [17]; this step generated a prediction of presence and absence of environmental conditions suitable for rabies occurrence.

We used two model performance measures designed for ENM predictions, based on external sets of validation areas and occurrences [17]. First, we used a cumulative binomial test to assess whether predictions of validation occurrences across the validation region were statistically significantly better than random expectations. We used the validation occurrences as number of trials, the number of validation occurrences correctly predicted as number of successes, and the proportion of pixels predicted as suitable by the model as the probability of a success [17,30]. Second, as a complementary validation of model performance, we used a modified version of the area under the curve (AUC) of the receiving operating characteristic (ROC), the Partial ROC [31], in which ROC curves are evaluated only over ranges of values that correspond to low omission errors. Here, performance was measured as a ratio between observed prediction and a random expectation (AUC = 1; e.g., *p* > 0.05), where ratios above 1 represent predictions better than random expectations (AUC > 1; e.g., *p* < 0.05), evaluated using a bootstrap test [31]. We used Partial ROC software [32] to develop these tests, with the following settings: 50% of occurrences for bootstrap, 1000 permutations, and a threshold of 95% occurrences successfully predicted (for a detailed explanation, see [33]).

### Model calibration and validation (environmental space)

We also validated the model in the environmental space, using environmental and occurrence datasets spatially and temporally independent of those used for model calibration (see Figure 3). A rabies’ ENM calibrated in one region and period was transferred and analyzed in environments used to calibrate another rabies’ ENM from a different area and period, and the shape, position, and size of both ENMs were compared to assess if our method was able to capture rabies’ environmental signature across different times and geographic areas. This novel approach for model validation was developed using the software NicheA version 3.0, a powerful tool for display and analysis of ecological niches in environmental space [34]. First, we generated a model for the calibration area (see above and Figure 3) by calculating the minimum-volume ellipsoid including the occurrences for calibration from 2002–2011 against corresponding environments. We transferred this model to the validation area, occurrences, and environments (Figure 3) for 2012 to assess the predictive ability of the calibration model. We measured the proportion of overlap of the two ellipsoids for the two environmental data sets, as an estimation of niche similarity and robust prediction among areas and time frames.

### ENM projection across Chile

After validating model predictive accuracy in space and time, we generated a country-wide model. NDVI datasets for January 2002 - December 2012 (i.e., the complete study period) were grouped by season using specific dates (see above). We used all available occurrences to generate the countrywide model.

Rabies occurrence records in Chile are influenced by biases introduced by passive surveillance [19], and this bias impacts model accuracy [35]. In this context, geographic bias correction has been suggested as an useful step in ENM [36]. Another factor affecting model output is the study area extent: the smaller the study area relative to the distribution of the species, the greater the challenge for the algorithms to produce accurate niche models [16]. In a previous study, Escobar et al. [37] found that introducing sampling effort in the form of number of rabies samples submitted reduced uncertainty, but did not improve model performance in terms of area predicted. Thus, to consider the bias sampling during our model calibration, we focused on sampled areas instead of number of bat samples from surveillance. We calibrated models in the municipalities of central Chile known to be rabies positive according to surveys. Once the final model was validated and calibrated in these specific areas, we transferred it to the whole country.

### Mapping human risk of rabies infection

To assess risk of human cases, we combined the binary map (presence/absence) of environmental suitability for rabies with a surface of human population. To establish current human population at risk of rabies transmission at fine resolutions, we filtered the rabies potential distribution map by location of human settlements. The distribution of human settlements was derived from mean night-time light satellite imagery by county, as this variable has a strong association with density of human populations [38]. We calibrated our population density estimates by regressing country-averaged night-time light values against human densities from the 2012 census data for Chile [39] by county area (km^2^). We used imagery at ~0.75 km resolution, specifically the band that detects light from visible to infrared acquired for 9 days in April and 13 days in October 2012 by the VIIRS sensor on the Suomi NPP satellite [40]. We used the first of the three bands that compose this image, with pixel values ranging 0–255: low values indicating darkness and high values indicating artificial light from human settlements. Although some night-time lights captured by the satellite could be wildfires or lit areas without human residents, we assumed that most values were related to artificial light.

After calibrating the satellite data against census reports, we classified light values into three categories, based on a quantile approach commonly used as a pragmatic criterion by which to define low, medium, and high risk of exposure [41]. The first quantile represents low risk, because few people are exposed in those areas; the second quantile is moderate risk; and the third quantile is high risk, as this class includes rabies-suitable cells with highest human density. To validate whether points falling in specific night-time light values could predict the human density category assigned to that light value, we generated 350 random points across the study area, and used a cumulative binomial test with the number of random points as trials, points with correct prediction of light value and human density as successes, and 1/3 (the chance of falling in the correct human density category) as the probability of a success.

## Results

### Validation of input data

A total of 353 bat-rabies occurrences during 2002–2011 overlapped with the calibration area (see Additional file 1). When models were transferred to the validation area, only one of 46 validation occurrences from 2012 was not predicted successfully (Additional file 1); this was statistically better than random predictions (*P* < 0.001; Table 1). This result indicated good performance of Maxent models across time, geographic areas, and environments. The most informative variables in model calibration were mean NDVI values for winter and spring, followed by maximum and minimum values in winter; least informative were minimum NDVI values for fall and summer.

**Table 1:** **Statistical validation of rabies model performance using external occurrences and validation areas, Chile, 2002–2012**

	**Cumulative binomial test**					**Partial ROC**
	**Pixels predicted present**	**Pixels predicted absent**	**Number of occurrences predicted present**	**Number of occurrences predicted absent**	**Binomial probability**	**AUC ratio min - max** ^*^
Validation of input data	20 859	61 458	45	1	5.09 × 10^−23^	1.52 - 1.87
ENM projection across Chile	15 532	60 630	113	27	1.10 × 10^−52^	1.10 - 1.62

^*^min = minimum AUC ratio from 1000 permutations.

^*^max = maximum AUC ratio from 1000 permutations.

When occurrences from calibration and validation areas (see Figure 3) were displayed in environmental space, they showed high overlap (Additional file 2). The NicheA algorithm uses geographic occurrences to collect environmental values, thus, analysis are based on environmental values only, excluding the geographic coordinates; this allowed us to display occurrences from different calibration and validation areas and distinct NDVI periods in a common environmental space (Figure 3 and Additional file 2). The ellipsoid of the occurrences and environments from 2002–2011 data in calibration regions was 35% larger compared to the volume of the ellipsoid of the occurrences and environments in validation areas, from 2012 (green ellipsoid in Additional file 2). Thus occurrences from the calibration areas encompassed larger environmental variation than those available in the validation areas (Additional file 2), which makes sense in light of the greater latitudinal diversity in the calibration subsets. These model validation exercises support the idea that selected environmental variables, study area delimitation, and available occurrences allow us to generate a robust ENM at a national extent.

### ENM projection across Chile

To generate a high-confidence bat-borne rabies risk map at the country extent, we examined environmental variables across the country. Large geographic areas showed low variations in NDVI values across seasons: a large number of cells had consistently low NDVI values (500–1500), reflecting arid conditions in northern Chile, while the number of cells with very low (water) or very high (forest) NDVI values varied seasonally. The validation occurrences were predicted correctly by the model better than chance expectations, with all AUC ratio values above 1.0 (*P* < 0.001; Table 1). Considering the robust model validation results, we developed a niche model using all occurrences and all positive counties, and transferred model rules to the entire country (Additional file 3). The resulting model identified approximately 25 000 km^2^ suitable for bat-borne rabies occurrence, concentrated in coastal areas of central Chile.

### Mapping human risk

**Figure 4:**
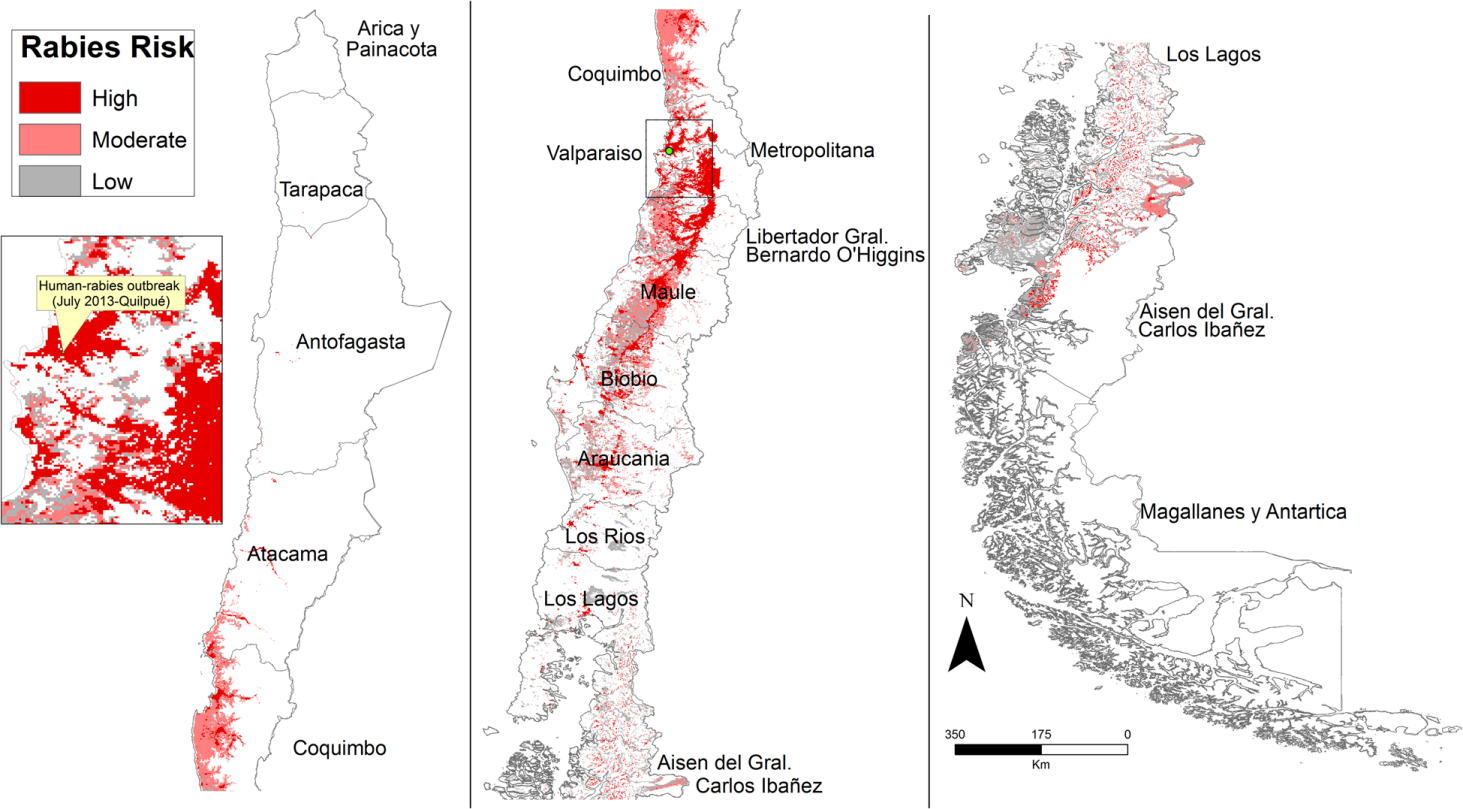
**Classification of areas at low (gray), moderate (pink), and high (red) risk of rabies transmission to humans.** Political boundaries at state level (region) are labeled for the northern (left panel), central (middle panel), and southern Chile (right panel). Insert: the last case of human rabies reported in Valparaiso in July, 2013 (green point).

We found a strong non-linear association between human density and average night-time light intensity by county (*r*^2^ = 0.83, *P* < 0.001; Additional file 4). Consequently, we used this imagery to classify potential distribution of rabies obtained through ENM into areas of low (0–4 people/km^2^), moderate (4–10 people/km^2^), and high (10–255 people/km^2^) risk. As a result, ~8600 km^2^ were classified as high risk (Figure 4). Large areas of high risk were located close to the foothills of the Andes Mountains (Figure 4). During the development of this study, the first case of human rabies reported in the last 17 years was confirmed in Quilpue county, Valparaiso region, in an area classified as high risk with our methodology (Figure 4).

## Discussion

The most densely populated areas, in central Chile, are suitable for bat-borne rabies. We identified a subset of four environmental variables (from seasonal NDVI data) that contained most of the information by which to predict rabies distribution at the national scale. These four variables summarize approximately 2400 days of satellite data compilation. Ecological niche models generated using few climatic variables usually generate broad geographic predictions, however, our models avoided overprediction, perhaps thanks to the heterogeneous values across the study area and reduced spatial autocorrelation provided by NDVI [42].

Using 12 NDVI layers, we found that NDVI values in winter have a particular impact in model performance. Previously reported evidence of reduction in rabies reports in the winter season in Chile [19] could reflect migration or decrease in bat activity. Characterization of vegetation values that shape aspects of the species’ niche in environmental space could be used to predict changes in species geographic distributions once vegetation changes occur, for example anticipating disease emergence after landscape disturbance (e.g., logging). To our knowledge, only one previous study explored the ability of ENM to anticipate disease occurrence using different time periods of vegetation indices over different geographic areas [43]: potential distribution of West Nile virus (WNV) in central United States was evaluated using vegetation indices and disease occurrence for 2002 and 2003, demonstrating that models calibrated in different geographic areas and time periods could anticipate human cases of WNV.

### Ecological niche modeling in epidemiology

Linking species’ distributions to environmental factors has been an important goal in ecology for a century [44]. Recent developments in the field of ecological niche modeling have provided conceptual bases to select algorithms, delimit study areas, evaluate models, and identify relevant environmental variables [16,45-50]. This study applied theory and methods from the field of ecology into epidemiology to map potential bat-borne rabies risk.

Current methods to generate risk maps of infectious diseases are usually based on disease-occurrences density. This approach estimates risk based solely on spatial interpolations [51], but such estimation may fail to anticipate risk in neglected areas (i.e., lack of surveillance) [52] or may assume high risk in oversampled areas [51]. On the other hand, we argue that maps based on environmental interpolations to model the pathogen’s ecological niche provide more accurate, and biologically realistic, predictions [53]. This ecological approach offers the opportunity to identify areas where the pathogen could be more abundant and genetically diverse based on suitable conditions [54,55]. Strikingly, despite its robustness, ecological niche modeling is still under-exploited in epidemiology [56].

ENM applications to infectious diseases are generally more complex than biodiversity studies [15]. Disease systems usually include several organisms: pathogens, vectors, natural reservoirs, and potential terminal hosts (e.g., humans), making application of ENM dependent on the target organisms, and on temporal and spatial scales [15]. The rabies system is clearly an example of spatial dependence and variation of environmental factors required by the virus for its persistence. At a very fine, sub-organismal scale, rabies virus shows affinity to the nervous system compared to all tissues available in the host, but with special preference for the brainstem and medulla [1]. At the other extreme of the spatial scale (i.e., continental), rabies responds to climate [14], reflecting how environments relate with all the participants in the system (the black box *sensu* Peterson [13]). Our previous exploration of ecological niche similarities between host and virus in the rabies system suggests that co-evolutionary forces may explain the close interaction between both organisms, represented in the indistinguishable occupancy of the environmental space by host and virus when explored at landscape scale [37]. Thus, species occupying the niche of another species (e.g., a parasite) may generate indistinguishable niche models between both species (i.e., the parasite and the host). Modeling rabies and bats generates indistinguishable niches, but modeling solely rabies provides more detail in the areas predicted suitable, gaining the prediction of risk [37].

Here, we focused our modeling at intermediate spatial resolution, fitting the spatial extent and available occurrences to remote sensing variables, but models at finer resolutions should be explored (e.g., free-ranging dog density, human behavior, bat abundance) to evaluate risk of rabies spillover in Chile. An area of ~8600 km^2^ was classified as at high risk of rabies occurrence, representing 1.1% of the total area of Chile (756 096 km^2^). This result provides a useful identification of priority areas.

### Model validation

Our validation design using different calibration and validation areas ensures statistical independence [57]. Validation is a crucial step in spatial epidemiology, especially for virulent pathogens such as rabies [42,56]. Unfortunately, in many applications of ENM to disease systems, validation procedures have been weak or even lacking [58-61]. Our assertive anticipation of rabies cases in independent validation areas is an example of how ENM of rabies may forecast rabies occurrence in unsampled areas, once the model is transferred to areas that lack data. Under the same approach, model transfer could be applied to future land-use change scenarios to predict rabies distribution under different NDVI values. The idea of an increase in rabies spread due to land-use change was proposed in previous reports of rabies in vampire bats [62-64], but has not been explored quantitatively in much detail until now. Our conservative model transfer settings avoided extrapolation into novel environmental conditions while allowing robust predictions into distinct NDVI datasets (i.e., 2012). Using extrapolation in ENM exercises generates perilous overprediction [29].

No set rules exist for selecting validation areas and occurrences. Rather, selection should be based on careful consideration of the data available and the biogeographic features of the accessible area selected *a priori* for the target species [17,65]. Selection of validation occurrences outside of areas used for model calibration (e.g., Figure 3) increases the geographic independence of validation occurrences from those used for calibration [42]. Model calibration should be developed using only areas with known disease occurrence, as this offers a means of reducing sampling bias, allowing detailed characterization of environments for rabies occurrences and avoiding uncertainty from non-sampled areas [35]. We calibrated the final model only in municipalities with known reports of rabies occurrences and found that focusing ENM calibration only in areas with surveillance reduces over-prediction and increases model accuracy (Additional file 3), albeit potentially at the cost of precision [36]. Consideration of sampling bias is an issue of critical relevance when modeling the niche of pathogens. Our preliminary exploration of model response when negative samples were added to the analysis showed that models reduced their variability, but without significant improvement of areas predicted [37]. Thus, bias from the geographic space (e.g., clustered occurrences), should be considered different from the bias from the environmental space. To make it simple, we could have an intense and uniform sampling effort across a large area, but the environmental representation may be minimal (e.g., thousands of occurrences in a desert could represent a single environmental value). On the other hand, we could have a low geographic coverage (i.e., few occurrence points), but these points capture the complete pattern of environmental signature that shapes the species distribution. In our study, even though rabies occurrences from urbanized areas were abundant, occurrences from non-urbanized areas allowed us to generate models with high predictive performance (Figure 3, Additional files 1 and 2). However, a well-designed sampling effort may provide more environmental information from areas not available to us, generating models with broader areas predicted suitable for bat-borne rabies occurrence.

In summary, our validation experiments confirmed that the ENM used in epidemiology can produce a robust risk map of a dangerous disease when occurrence data quality, environmental variable manipulation, and study area extent are considered carefully. As a result of these analyses, government efforts to ward prevention and control can focus in geographic areas most suitable for rabies potential distribution.

## Additional files

Additional file 1:
**Validation of rabies prediction for central Chile in 2012, generated based on data from 2002–2011 in northern and southern Chile.** Occurrences and environments from 2002–2011 (yellow squares) were used for model calibration (black scale). The model transferred to 2012 environments (dashed line; validation area) showed correct prediction of occurrences from 2012 (green points), with one exception (black triangle). Additional file 2:
**Niche models from different areas validated in environmental space using NicheA.** Calibration occurrences, areas, and environments (red; calibration in Figure 3) and validation occurrences, area, and environments (green; validation in Figure 3) are displayed in the environmental space. Background is represented by NDVI values for 2012 (gray points). Notice that the two ellipsoids overlap completely (100%). Additional file 3:
**Ecological niche modeling using occurrences and seasonal NDVI values from all data available (2002 to 2012).** The model was calibrated in all positive counties (blue polygons), and transferred to a national extent (left) to estimate the rabies potential distribution, shown in red. Additional file 4:
**Association between human density and night-time light values.** Values of mean night-time light values (0–255) and human population (people/km^2^) by county (black circles). Our local polynomial regression model (LPR) estimated values shown as red circles (*r* ^*2*^ = 0.83; *P* < 0.001). Night-time light imagery has been used to describe features of human settlements such as human density and social and economic parameters [66-68]. Despite open access to these data and powerful software available for their analysis, exploration of this source of information is scarce. A notable exception is a recent study of measles [69]. However, it must be borne in mind that in epidemiology, night-time light data have several limitations: (i) application of night-time light imagery is temporally and spatially dependent, such that its use must include validation of predictive relationships to variables of interest in each study case; (ii) light values in remote areas may be the product of fire, biasing estimates of human settlements; (iii) industrial areas with high human density may show low light values at night, underestimating numbers at risk; and (iv) models using remote sensing data to estimate human population may overestimate areas with low population, which may indicate that very isolated light spots are unreliable predictors of population. To correct the latter, some techniques include delimiting urban areas [70], but such methods miss the point of our risk classification.
