# Supplementary figures and images for: Ecological approaches in veterinary epidemiology: mapping the risk of bat-borne rabies using vegetation indices and night-time light satellite imagery
Source: Vet Res. 2015 Sep 4;46(1):92. doi: 10.1186/s13567-015-0235-7 (PMC4558958; doi:10.1186/s13567-015-0235-7)

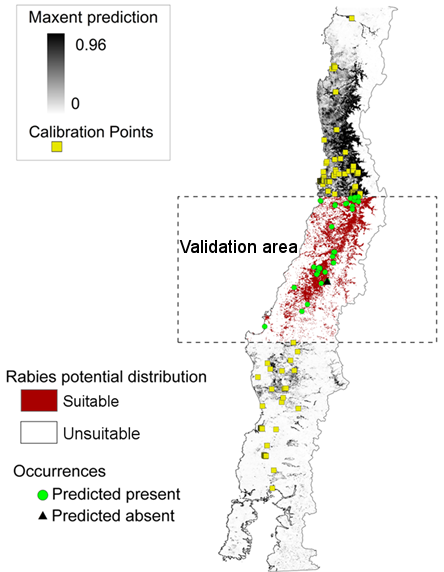

Supplement: Additional file 1: — Validation of rabies prediction for central Chile in 2012, generated based on data from 2002–2011 in northern and southern Chile. Occurrences and environments from 2002–2011 (yellow squares) were used for model calibration (black scale). The model transferred to 2012 environments (dashed line; validation area) showed correct prediction of occurrences from 2012 (green points), with one exception (black triangle). [file 13567_2015_235_MOESM1_ESM.tif]

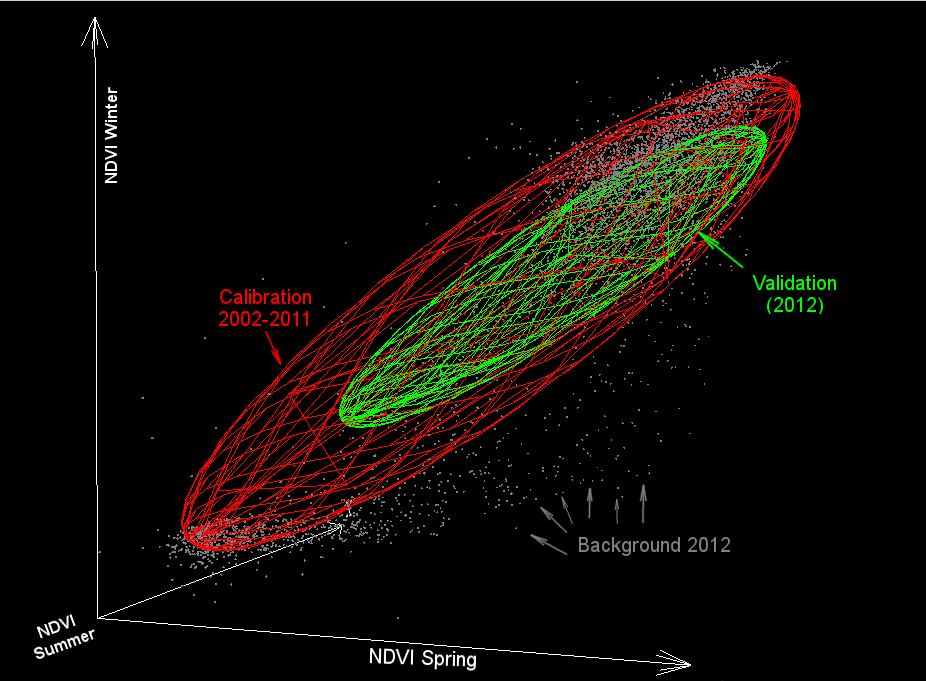

Supplement: Additional file 2: — Niche models from different areas validated in environmental space using NicheA. Calibration occurrences, areas, and environments (red; calibration in Figure 3) and validation occurrences, area, and environments (green; validation in Figure 3) are displayed in the environmental space. Background is represented by NDVI values for 2012 (gray points). Notice that the two ellipsoids overlap completely (100%). [file 13567_2015_235_MOESM2_ESM.tif]

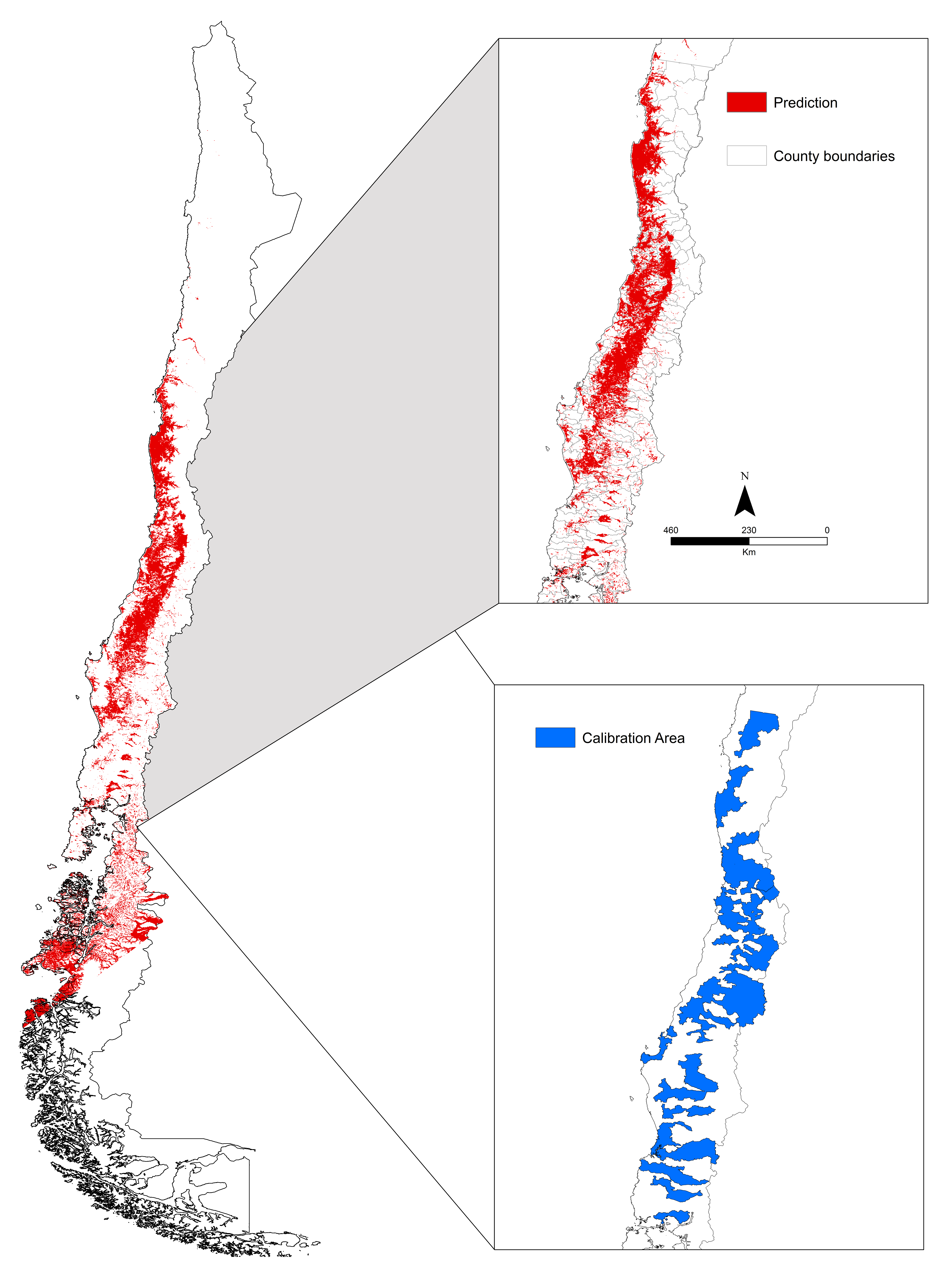

Supplement: Additional file 3: — Ecological niche modeling using occurrences and seasonal NDVI values from all data available (2002 to 2012). The model was calibrated in all positive counties (blue polygons), and transferred to a national extent (left) to estimate the rabies potential distribution, shown in red. [file 13567_2015_235_MOESM3_ESM.tif]

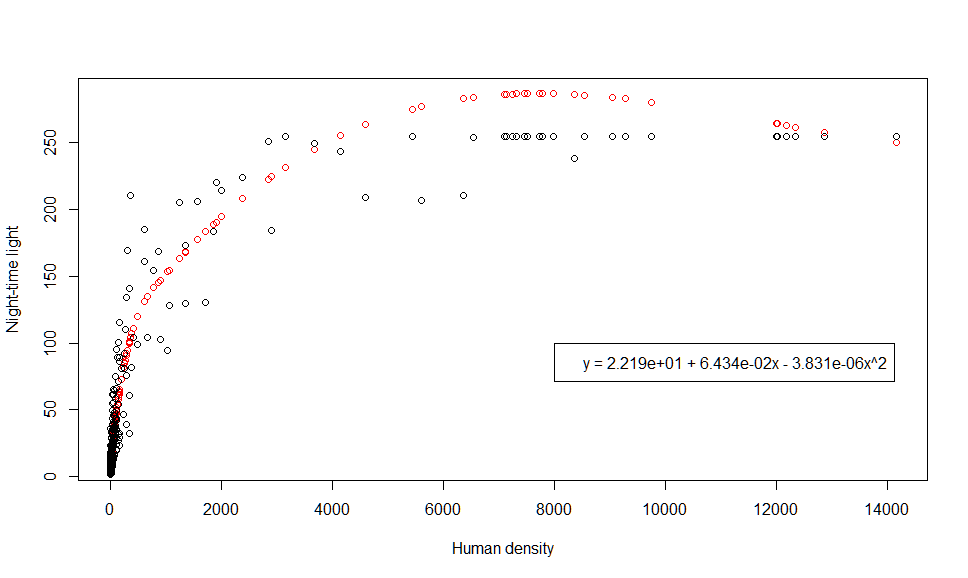

Supplement: Additional file 4: — Association between human density and night-time light values. Values of mean night-time light values (0–255) and human population (people/km2) by county (black circles). Our local polynomial regression model (LPR) estimated values shown as red circles (r 2 = 0.83; P < 0.001). Night-time light imagery has been used to describe features of human settlements such as human density and social and economic parameters [66-68]. Despite open access to these data and powerful software available for their analysis, exploration of this source of information is scarce. A notable exception is a recent study of measles [69]. However, it must be borne in mind that in epidemiology, night-time light data have several limitations: (i) application of night-time light imagery is temporally and spatially dependent, such that its use must include validation of predictive relationships to variables of interest in each study case; (ii) light values in remote areas may be the product of fire, biasing estimates of human settlements; (iii) industrial areas with high human density may show low light values at night, underestimating numbers at risk; and (iv) models using remote sensing data to estimate human population may overestimate areas with low population, which may indicate that very isolated light spots are unreliable predictors of population. To correct the latter, some techniques include delimiting urban areas [70], but such methods miss the point of our risk classification. [file 13567_2015_235_MOESM4_ESM.tiff]
